# Progress of 3D Bioprinting in Organ Manufacturing

**DOI:** 10.3390/polym13183178

**Published:** 2021-09-18

**Authors:** Dabin Song, Yukun Xu, Siyu Liu, Liang Wen, Xiaohong Wang

**Affiliations:** 1 Center of 3D Printing & Organ Manufacturing, School of Intelligent Medicine, China Medical University (CMU), No. 77 Puhe Road, Shenyang North New Area, Shenyang 110122, China; songdabin1105@163.com (D.S.); xuyukun1106@163.com (Y.X.); 17824224369@163.com (S.L.); lwen@cmu.edu.cn (L.W.); 2 Key Laboratory for Advanced Materials Processing Technology, Department of Mechanical Engineering, Tsinghua University, Ministry of Education & Center of Organ Manufacturing, Beijing 100084, China

**Keywords:** polymers, biomaterials, 3D bioprinting, organ manufacturing, cells

## Abstract

Three-dimensional (3D) bioprinting is a family of rapid prototyping technologies, which assemble biomaterials, including cells and bioactive agents, under the control of a computer-aided design model in a layer-by-layer fashion. It has great potential in organ manufacturing areas with the combination of biology, polymers, chemistry, engineering, medicine, and mechanics. At present, 3D bioprinting technologies can be used to successfully print living tissues and organs, including blood vessels, skin, bones, cartilage, kidney, heart, and liver. The unique advantages of 3D bioprinting technologies for organ manufacturing have improved the traditional medical level significantly. In this article, we summarize the latest research progress of polymers in bioartificial organ 3D printing areas. The important characteristics of the printable polymers and the typical 3D bioprinting technologies for several complex bioartificial organs, such as the heart, liver, nerve, and skin, are introduced.

## 1. Introduction

**Figure 1:**
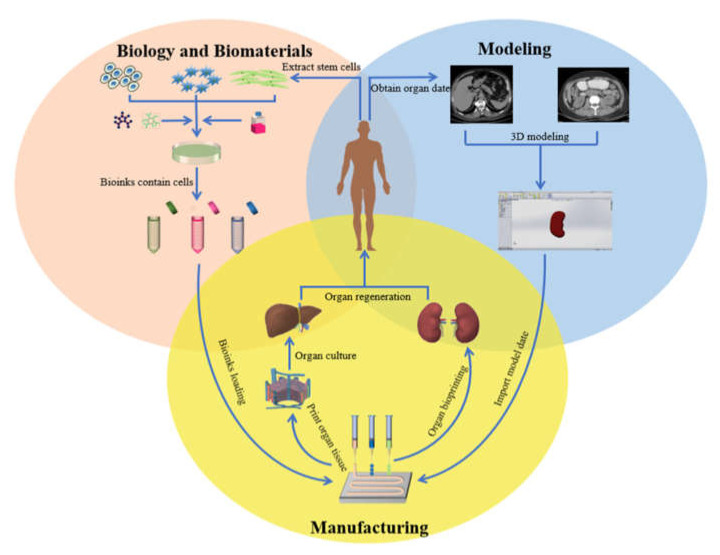
Organ 3D printing and application.

At present, the severe shortage of organ donor supply is a major problem that plagues the clinical medical community around the world. Organ manufacturing has become a major research topic for defective∕failure organs [1,2]. Organ manufacturing is a complex interdisciplinary field that requires a large number of talents in biology, materials, chemistry, physics, mechanics, informatics, computer, medicine, etc. It is also a compositive process that requires model construction, biomaterial selection, and the combination of multiple cell types along with other biomaterials using advanced processing technologies [3,4,5] (Figure 1). The program to form a functional structure is a key step for the construction of multiple cell types with hierarchical vascular/neural/lymphatic networks in a biological artificial organ (i.e., bioartificial organ).

Three-dimensional (3D) bioprinting technology is traditionally termed as rapid prototyping (RP), additive manufacturing (AM), and solid free-form manufacturing (SFM). It can produce bioartificial organs through automatic layer-by-layer deposition methods [6]. The most obvious feature of 3D bioprinting technology is the use of living cells, polymeric hydrogels, and other bioactive agents as “bioinks” to construct bioartificial organs under the instruction of a computer-aided design (CAD) model [7,8]. Multiple cell types can be encapsulated in different polymeric hydrogels and deposited (or delivered) at the same time. Under certain biological/physical/chemical conditions, hydrogels can absorb and retain a large amount of water, which is beneficial for cell growth, proliferation, differentiation, and the formation of tissues/organs [9,10,11].

**Figure 2:**
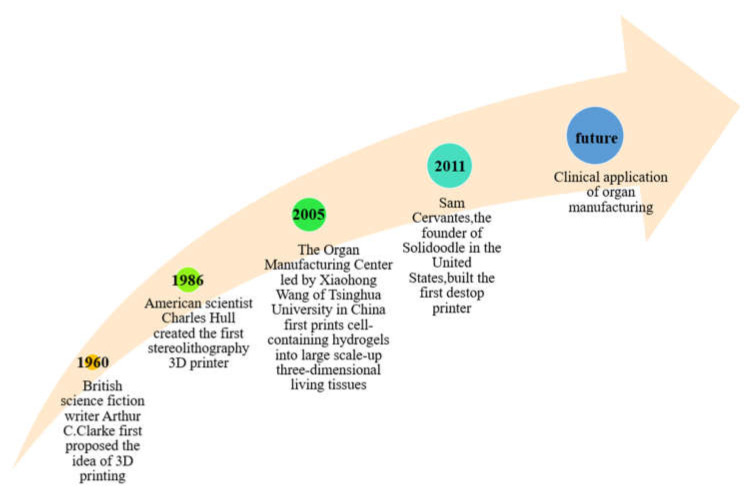
Historical events of 3D printing and organ 3D bioprinting.

The properties of polymeric hydrogels are very important in organ 3D printing. Polymers can not only provide a template for cells to grow, proliferate, and differentiate, but also supply nutrients and discharge metabolites for cells in the constructs. For organ manufacturing, it is normally necessary for the polymers to be biocompatible and biodegradable with adjustable mechanical properties (Figure 2) [12,13,14,15].

In this review, we give a comprehensive overview of the latest advances in organ 3D bioprinting using various polymers. The unique advantages of 3D bioprinting technology in the field of organ manufacturing have been highlighted.

### 2. 3D Bioprinting Technologies

**Figure 3:**
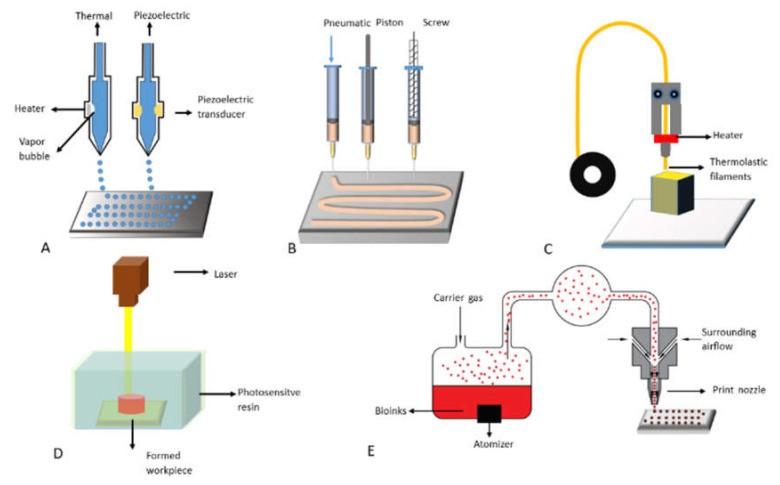
Commonly used 3D bioprinting technologies: (**A**) inkjet-based 3D printing; (**B**) extrusion-based 3D printing; (**C**) fused deposition modeling (FDM); (**D**) stereolithography; (**E**) aerosol jet printing.

During the last decade, 3D printing technologies have developed rapidly and have been applied to almost every biomedical field [16]. Compared with traditional manufacturing technologies, these technologies have many advantages in bioartificial organ manufacturing, especially for fast, precise, and customized biomedical applications. In Table 1, we have compared the advantages and disadvantages of several commonly used 3D bioprinting technologies for bioartificial organ manufacturing (Figure 3). The construction of hierarchical vascular and neural networks has been emphasized.

#### 2.1. Inkjet-Based 3D Printing

Inkjet-based 3D printing is a non-contact AM technology adapted from industrial 2D printers. In traditional 2D printing, a layer of droplets are selectively deposited, while in 3D bioprinting, cells and proteins can be printed into desired shapes of an organ by changing the content of “bioinks” and deposition layers. Drop-on-demand inkjet printers are the most commonly used equipments, consisting of acoustic, thermal, piezoelectric, or electrostatic inkjet nozzles [17]. Inkjet printers are usually employed to print tissue engineering scaffolds for cell seeding. Recently, different inkjet printing heads with multiple nozzles have been developed to improve the printing speed and structural size [18]. Although this printing method has some advantages in organ manufacturing, poor printing resolution and low printing speed have greatly limited its fidelity and clinical implementation. Until now, only simple structures with few layers of “bioinks” can be achieved. For example, Gao [19] et al. developed a bioprinting platform for 3D cartilage tissue engineering using thermal inkjet printers and polyethylene glycol (PEG)-diacrylate (PEGDA, MW 3400). Using this platform, the precise distribution and arrangement of human mesenchymal stem cells (h MSC) can be realized. After printing, the cells in the 3D PEGDA hydrogel showed a chondrogenic phenotype with gradually increased glycosaminoglycans (GAG) and type II protein expression during the in vitro cultures, while the constructed cartilage-like construct showed a natural composition band organization and ideal mechanical properties. 

#### 2.2. Fused Deposition Modeling (FDM) 

FDM is a 3D printing method that deposits molten thermoplastic polymer layer-by-layer in a specific laying pattern through one or more heated extrusion heads with small holes [20,21]. It is also called “thermoplastic extrusion”. In FDM, one of the traditional methods is to melt the thermoplastic polymer into a semi-liquid state, and then extrude the semi-liquid polymer layer-by-layer onto the platform. The thermoplastic polymer before printing are generally in the form of filaments or particles. When the thermoplastic polymer is heated to a temperature above its melting point, it becomes fluid and flows out of the nozzle. As soon as the plastic polymer flows out of the nozzle, it hardens and bonds with the underlying layer. Once a layer is built, the platform is lowered or the nozzle is raised, the extrusion nozzle deposits another layer, and the printed layers are fused together in layers [20,21]. 

The often printed thermoplastic polymers include acrylonitrile butadiene styrene (ABS), polylactic acid (PLA), nylon/polyamide, acrylonitrile styrene acrylate (ASA), polyethylene terephthalate (PET), polyethylene terephthalate modified (PETG), polycarbonate (PC), polyether ether ketone (PEEK), polyether imide (ULTEM), and their derivatives, such as fiber reinforced composites and polymer ceramic composites [22]. The most important advantage of FDM technology in the AM field is that it can create complex scaffolds with excellent mechanical strength and high geometric accuracy. One of the fatal shortcomings of FDM technology in the field of organ 3D printing is that cells, growth factors, and other bioactive agents cannot be directly printed at high polymer melting temperatures. Nowadays, the 3D printing technology of FDM with PEEK as raw material is widely used in bone tissue engineering, orthopedic implants, joint replacement, spinal implants, prostheses, and dentistry [23,24,25]. A research team has used CT-guided FDM to manufacture a polycaprolactone (PCL)/hydroxyapatite bone scaffold with cortical bone-like characteristics, which is similar to natural bone in terms of structural features, mechanical properties, and chemical composition [26].

#### 2.3. Extrusion-Based 3D Printing

Similar to FDM, extrusion-based 3D printing deposits “bioinks” from a syringe or nozzle onto a build platform based on the CAD models [27]. Unlike FDM, the extrusion-based extrusion process does not involve any heating process unless it is particularly necessary. Polymer solutions or hydrogels with cells, growth factors, and other bioactive agents can be extruded through a nozzle in a controlled manner by pneumatic pressure or physical force (i.e., piston or screw). The deposition system is selected based on the sensitivity of the “bioinks”, with the purpose of gradually pushing them down without damaging the cells inside the “bioinks” [28]. Compared with other 3D printing processes, the extrusion-based 3D printing technology provides a higher printing speed. With the development of this 3D printing technology, multiple cell types can be deposited together with different biocompatible polymer solutions/hydrogels at very high cell density. The solidification of polymer solutions or hydrosols is through a series of physical and chemical methods, such as sol–gel conversion (i.e., physical crosslinking), polymerization, chemical crosslinking, and enzymatic reactions [29].

In some extrusion-based 3D printing experiments, the cell viability after printing is at the range of 40% to 80% [30,31]. The cell survival rate can be improved by optimizing the printing parameters, such as deposition rate, pressure, and temperature. Some researchers use gelatin methacrylamide loaded with cells, as “bioinks”, for printing, and the cell viability can be as high as 97% [32]. It was reported that adopting PEG cross-linking after 3D priniting can improve the mechanical properties of the 3D constuct while the cell viability is maintained [33].

In the past decade, extrusion-based printing has been the most widely used technology in the field of organ 3D bioprinting, and the products have been applied in many biomedical fields, such as high through-put drug screening, customized organ substitution, surgical assistance model preparation and pathological analyses. For example, Gu et al. [34] reported a reversible physical cross-linking strategy to accurately deposit gelatin methacrylic “bioinks” loaded with human chondrocytes at low concentrations without any sacrificial materials. Chen et al. [35] introduced 1% aldehyde hyaluronic acid (AHA) and 0.375% N-carboxymethyl chitosan (CMC) to obtain a polysaccharide gelatin (GEL, 5%)-alginate (ALG, 1%) “bioink”. This GEL-ALG/CMC/AHA “bioink” has a weak temperature dependence when it was printed in vivo at about 37°C using traditional printing methods. The printed cell-laden structure can have high underwater fidelity after being reinforced with 3% calcium chloride for only 20 seconds. The fidelity can be maintained for 30 days. 

Furthermore, in order to avoid the disadvantages related to the traditional extrusion-based 3D printing, more and more researchers are applying microfluidic techniqe into this group of technologies to produce biological structures. Beside producing tubular vascular structures, many elaborate vascular and neural networks have been obtained through the updated multiple nozzles or syringes [15].

#### 2.4. Stereolithography (SLA)

As the earliest 3D printing technology, SLA is the most mature 3D printing method and is widely used in the industry [36]. In SLA, a concentrated ultraviolet beam is irradiated on the liquid photopolymer, and the CAD model is interpreted to the liquid surface to start the building of the first layer. Subsequently, each layer plane polymerizes at a predetermined time to form a solid layer until the desired object is completely manufactured [37]. Compared with other traditional methods, light-curing biological 3D printing has the characteristics of high flexibility, high resolution, and fast manufacturing speed. These characteristics make it widely used in corneal stromal tissue regeneration, insulin delivery, tissue engineering scaffold manufacturing and other fields. Greatly promoted the progress in the field of tissue and organ regeneration. [38,39,40,41].

SLA has some advantages in tissue engineering areas, but its development and popularization in the field of organ manufacturing have been seriously restricted by many factors. (1) Printing technology limitations: different SLA technologies have different lamp wavelengths, printing sizes, and “bioink” viscosity requirements, which are hard to be standardized. (2) Material limitations: there are fewer photocurable biomaterials can be used as “bioinks” with the required low viscosity. (3) Cost restriction: the price of SLA instruments and their printed biomaterials are expensive, resulting in the limited clinical trials.

#### 2.5. Aerosol Jet Printing

Aerosol jet printing is another non-contact printing method with high resolution and flexibility. It has been developed recently, in which the “bioink” is placed in an atomization generator and atomized into aerosol particles via ultrasonic or pneumatic atomization, before being delivered to the print nozzle via inert gas. Inside the nozzle, a surrounding gas is applied to constrain the “bioink” before it was deposited on the surface of the substrate to form a functional pattern [42]. 

In aerosol jet printing, the “bioinks” can be printed on various substrates, such as metals, semiconductors, and polymers [43], and the products can be widely used in the field of electronics [44]. However, studies have shown that ultrasonic treatment can denature DNA [44], which has limited its application in the field of biomedicine. Until present, there are few studies on organ 3D printing using this method except heart patch engineering and protein detection [45,46].

## 3. Natural Polymers for Organ 3D Printing

Natural polymer refers to a high molecular weight compound whose basic structure is a linear long chain connected by repeating units that exists in animals, plants, and other organisms. Natural polymers include proteins, polysaccharides, and their combinations, such as glycoproteins, proteoglycans [47,48]. Most of natural polymers can dissolve in inorganic solvents, such as water and acetic acid. Water solvents under certain potential of hydrogen (pH) values are non-toxic to cells. Some natural polymer solutions, such as gelatin and agarose, can be cross-linked to form hydrogels with certain viscosity. These polymers usually have good biocompatibility and degradability and can meet the requirements for 3D bioprinting [49]. Living cells and bioactive agents can be embedded into the natural polymer solutions before 3D bioprinting. During the 3D printing process, the polymer chains can protect cells from squeezing pressures. After 3D printing, the 3D constructs can be stabilized by cross-linking the polymer chains, and nutrients and oxygen can be transported to the cells through the interpenetrating networks [50].

Currently, the most commonly used natural polymers for organ 3D printing are collagen, gelatin, alginate, fibrinogen, hyaluronic acid, chitosan, and agarose [50]. Each of these polymers has advantages and disadvantages in organ 3D bioprinting. In Table 2, the typical properties, advantages, and disadvantages of several commonly used natural polymers for organ 3D bioprinting have been summarized [51,52,53,54,55,56,57,58,59,60,61,62,63,64,65].

### 3.1. Gelatin

Gelatin is derived from collagen, obtained by partial hydrolysis and incomplete cleavage of collagen molecules [51,52]. Its water solution is a hydrocolloid with high freezing point, rapid degradation rate, low gel strength, and poor mechanical properties. As a natural polymer, gelatin has many advantages for organ 3D printing, such as excellent biocompatibility, physical gelation property, and low cost [66,67].

Gelatin can be printed with other natural polymers, such as chitosan, alginate, fibrinogen and hyaluronate [68,69,70,71,72,73,74,75]. There are several chemical cross-linking strategies for the stabilization of the gelatin-based 3D constructs during or after 3D printing. In Table 3, some commonly used gelatin-based composite hydrogels for organ 3D printing are summarized.

Due to its excellent biocompatibility and degradability, a gelatin derivative known as gelatin-methacryloyl (GelMA) has been widely explored for heart, cartilage, nerve, and other tissue 3D printing [76,77,78,79].

Some researchers used a pneumatic extrusion printer to precisely control the channel geometry of the 3D printed gelatin scaffold and seeded undifferentiated liver cell lines (Huh7) on the scaffolds. Compared to 2D cultures, hepatocytes in 3D-printed gelatin scaffolds were functionally increased in albumin secretion, cytochrome P450 oxidase activity, and bile salt transport activity [68]. Lam et al., printed methacrylated gelatin (GelMa) and methacrylated hyaluronic acid (HAMA) using a stereolithographic bioprinter, creating cartilage models with different chondrocyte densities. After 14 days in vitro culture, cells in the GelMA and HAMA hydrogels differentiated into a monolayer of chondrocytes, as evidenced by cartilage typical proteoglycan, cartilage specific type II collagen deposition, and cartilage marker gene expression. The technique can be applied to create cartilage repair models and treat articular cartilage defects using autogenous cells and compatible polymers with tailored sizes and shapes [69].

Gelatin prepared by a single modification sometimes cannot meet all the requirements for organ 3D printing. Many combinations and modifications have been exploited without increasing its toxicity to cells, enhance the mechanical properties, and stabilize the 3D printed constructs (Table 3) [76,77,78,79].

### 3.2. Alginate

Alginate is usually extracted from brown algae (Phaeophyceae), including kelp, Ascophyllum, and Macrocystis. Alginate solution is polyanion and has certain pH sensitivity. The higher the pH, the stronger the hydrophilicity of the solution, on the contrary, the weaker the hydrophilicity [53]. Sodium alginate hydrogel has good biocompatibility, strong gel-forming capability, low toxicity, low cost, and wide sources, which make it the most widely used natural polymer in organ 3D printing areas [54].

A sodium alginate molecule often has three parts, namely “M area (rich in mannuronic acid), “G area” (rich in guluronic acid), and “MG area” (containing mannuronic acid and Guluronic acid) [53]. Alginate solution has an important character that can be chemically crosslinked by divalent cations, such as calcium (Ca^2+^), strontium (Sr^2+^), and barium (Ba^2+^) ions. The crosslinked hydrogels have been applied in many biomedical fields, such as wound healing, drug delivery, and regenerative medicine [80,81,82].

The Na^+^ on the G unit can be ion-exchanged with divalent cations such as Ca^2+^, resulting in a large accumulation of G units to form a 3D network, which can quickly form a hydrogel. The preparation methods of sodium alginate hydrogel mainly include physical crosslinking, chemical crosslinking, and enzyme crosslinking. It is worth mentioning that the chemical crosslinking of alginate molecules using Ca^2+^ is reversible. When the 3D printed construct is placed in a liquid that does not contain or contains less Ca^2+^, the crosslinked Ca^2+^ will gradually dissolve within about one week.

As a natural polymer, there are fewer cell attachment sites in the alginate molecules, which makes it not the good candidate for cells to grow and proliferate inside the single alginate hydrogels. Thus, alginate is often used with other natural polymers, such as gelatin and fibrin. In organ 3D printing, the properties of the 3D printed costructs are often needed to adjust to meet the special requirements as regard to the water holding capacity, degradation rate, stability, and mechanical properties (e.g., rigidity) (Table 4).

A team used type I collagen, sodium alginate, and chondrocyte as 3D bioprinting “bioink” to construct cartilage tissues. The results show that the “bioink” has good mechanical strength and biological function, and it is very promising in cartilage tissue 3D bioprinting [83]. Another team used alginate/gelatin hydrogel to encapsulate aortic sinus smooth muscle cells (SMC) and aortic valve interstitial cells (VIC), and successfully printed aortic valve catheters. Both cell types achieved high survival rates in the 3D-printed constructs, indicating that aortic valve hydrogel catheters with complex anatomical structures and heterogeneous encapsulation can be 3D bioprinted with sodium alginate-based hydrogels [84]. Zohreh et al., used sodium alginate/chitosan/hesperidin hydrogel to print bioartificial skins with good biocompatibility, mechanical properties, and antibacterial properties. In the full-thickness rat skin trauma repair experiment, the hydrogel had a better wound closure effect than the gauze-treated wounds [85]. Researchers, such as Ning, used a composite hydrogel of alginate, fibrin, hyaluronic acid, and (Arg-Gly-Asp, RGD) peptides to encapsulate Schwann cells to print 3D bio-scaffolds, and applied them to neural tissue engineering. Histological experiments showed that Schwann cells in the constructs had high viability, proliferation rate, and protein expression level. It shows that the alginate-based mixture can be used to produce nerve repair scaffolds, and these scaffolds have great potential in the field of post-peripheral nerve regeneration [86].

### 3.3. Fibrin

**Figure 4:**
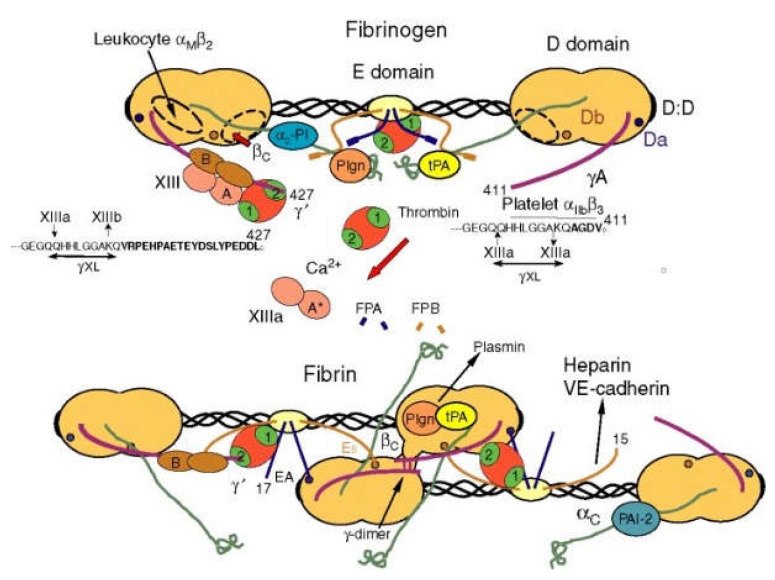
Schematic diagram of fibrin transformed from fibrinogen [55].

The fibrinogen molecule is composed of two sets of alpha, beta, and gamma chains bridged by disulfide bonds. Each molecule contains two outer D domains, which are connected to the central E domain by a coiled-coil segment (Figure 4). Fibrin is formed after thrombin cleaves fibrinopeptide A from the Aα chain of fibrinogen, which initiates the polymerization of fibrinogen [55]. Fibrin has super biocompatibility and biodegradability. Fibrin gel is non-toxic and does not have immune rejection [56], so it is widely used in the liver and heart 3D printing areas [57].

Fibrin was first printed into large living tissues in 2007 [70,71,72,73,74,75]. Since then it has been utilized for ogan printing with many other natural and synthetic polymers. Some researchers used a multi-nozzle 3D printer to print gelatin/alginate/fibrinogen hydrogel scaffolds, and a 3D biological printing glioma stem cell model was established. The survival rate of glioma stem cells in the scaffold was 86.92%, and the cells have high viability and differentiation potential in this hydrogel scaffolds [100]. A team used fibrin as bioinks and an extruded biological 3D printer to print fibrin scaffolds containing human fibroblasts, and inoculated keratinocytes on the scaffolds to obtain human double-layer skin. Histological results showed that the structure and function of printed skin were similar to human skin. The results show that fibrinogen can be used to print artificial skin [101]. 

### 3.4. Chitosan

Chitosan is a derivative of chitin obtained by deacetylation of chitin. Its chemical name is polyglucosamine (1-4)-2-amino-B-D glucose [63]. Chitosan has average biocompatibility, biodegradability, and non-toxicity in relation to cells and tissues [64].

Ducret et al. combined fibrin and chitosan to design an injectable hydrogel to determine the antibacterial effect of chitosan in fibrin hydrogel, as well as the vitality and proliferation state of dental pulp (DP)-MSCs in the hydrogel. It shows that the fibrin–chitosan hydrogel has strong antibacterial capability and can promote the regeneration of human DP tissue through maintaining a bacteria-free environment in the DP space [102]. Demirtaş et al. compounded chitosan with nanostructured bone-like hydroxyapatite to prepare a chitosan–hydroxyapatite hydrogel. MC3T3-E1 pre-osteoblasts were loaded with chitosan and chitosan–hydroxyapatite hydrogels, and printed with an extrusion 3D bioprinter. The results showed that chitosan and chitosan–hydroxyapatite can provide good mechanical support for 3D bioprinting. Cells in the hydrogels can maintain viability and undergo proliferation and osteogenic differentiation [103]. Wang’s team constructed a new type of collagen/chitosan/heparin matrix and found that it is highly porous and can be stabilized at least 60 days in vitro in a PBS buffer solution containing collagenase/lysozyme at 37 °C. The hydrogel-cultured hepatocytes showed high urea and triglyceride secretion function 25 days after inoculation, indicating that the collagen/chitosan/heparin matrix has great potential in the field of liver manufacturing [104].

### 3.5. Agarose

Agarose is a linear polymer, and the basic structure is 1,3-linked β-D-galactose and 1,4-linked 3,6-lactose-L-galactose alternately linked long chains. Agar pectin is a heterogeneous mixture composed of many smaller molecules. Agarose is generally heated to above 90 °C to dissolve in water, and a good semi-solid gel is formed when the temperature drops to 35-40 °C. This is the main feature and basis for its multiple uses. As a natural polysaccharide material, it has the characteristics of cell non-toxicity, slow degradation rate [105], low material cost, and has good mechanical properties after gelling [65].

Agarose has been widely used in hard tissue repair scaffolds, wound dressings, drug delivery, and other fields. Sivashankari et al. used freeze-drying technology to prepare a 3D porous scaffold of agarose/chitosan/graphene oxide (ACGO), and evaluated the physical, chemical, and biological properties of the composite scaffold. The connected holes have good blood compatibility and Vero cell proliferation ability, indicating that the ACGO composite scaffold can be applied to tissue engineering applications [106]. Carriel et al. developed a human skin replacement model using fibrin–agarose hydrogels, and evaluated the model in vivo and in vitro through histological experiments. The fibrin–agarose hydrogels can reproduce the histological structure of natural human skin [107]. Junji et al. developed a hydroxyapatite/agarose gel and printed it into 3D scaffolds. The 3D scaffolds were implanted into the medial femoral condyle of rabbits. After implantation, the bone regeneration process was evaluated by micro-focus computed tomography (micro-CT) and histological analysis. New bone was seen at the edge of the bone defect 2 weeks after the operation, the bone regeneration was good at 4 weeks after the operation, and the implant gradually degraded 8 weeks after the operation [108].

## 4. Synthetic Polymers for Organ 3D Printing

Synthetic polymers are man-made polymers with adjustable chemical structures and physical properties produced by chemical reactions. Most synthetic polymers have better mechanical properties than natural polymers. However, synthetic polymers are biologically inert and the printing process usually involves the use of organic solvents and/or toxic activators, which may reduce the biological effects of cell activity. Therefore, biologically active ingredients (such as cells and growth factors) cannot be easily combined with synthetic polymers for 3D printing. Since most synthetic polymers can be degraded by microorganisms or biological fluids in the body [109,110], the degradation rate can be adjusted to match the generation rate of specific tissues and organs. The commonly used biodegradable synthetic polymers in 3D printing include poly (lactic acid) (PLA), poly (glycolic acid) (PGA), polylactic-co-glycolic acid (PLGA), polyurethane (PU), and polycaprolactone (PCL) [111,112,113,114,115,116]. In Table 5, some of the characteristics for organ 3D printing are summarized.

Because they can withstand the internal and external tension of the 3D printing process and the implantation stage in the body and have good mechanical strength, synthetic polymers have a priority in the field of hard tissue and organ 3D printing [117,118,119].

For organ 3D printing, synthetic polymers are often used together with natural polymers. Especially, for large-scale hierarchical structures, such as layered blood vessels, nerve tissues, and lymphatic networks, synthetic polymers prefer to be the wrappage, wrapping outside the cell-laden natural polymers [120,121].

### 4.1. PLA

As a linear thermoplastic aliphatic polyester, PLA is mainly synthesized from starch raw materials through saccharification, fermentation, and other chemical reactions. PLA has good biocompatibility and biodegradability. It can be completely degraded under certain conditions. The final metabolic products are carbon dioxide and water, which can be eliminated from the bodys [122,123,124]. PLA has many advantages in organ 3D pringing, such as good thermal stability, water/solvent/bacteria resistance, excellent gloss, transparency, and flame retardancy [125].

Since 3D printing can control the overall geometry and internal structure of the 3D constructs, it has become a key process for hard tissue and organ, such as bone and cartilage, substitute manufacturing. The PLA based synthetic polymers therefore have become the the main focus for large size bone and cartilage scaffold development. The hybridization of PLA with natural polymers has become a promising method to manufacture complex organs [134]. Due to the excellent mechanical properties, biocompatibility, and degradability of PLA, it has been widely used in the related research. For example, Chen et al. have uniformly dispersed nano-hydroxyapatite to PLA and fabricated a 3D composite scaffold with enhanced osteogenesis and osteoconductivity through a desktop FDM technology. The 3D printed PLA/nano-hydroxyapatite scaffold has great potential in repairing the large bone defects. [135]. Shadi et al. used an indirect 3D printing method to develop a bone repair scaffold made of PLA/PCL/hydroxyapatite with macropores and micropores. The composite scaffold with a weight ratio of 70/30 (PLA/PCL) achieves more favorable properties in terms of biocompatibility and osteoinductive properties [136].

### 4.2. PLGA

PLGA is a synthetic copolymer (polyester) of lactic acid (LA) and glycolic acid (GA) with excellent physical, chemical, thermal, and mechanical properties that makes it suitable for wide usage. PLGA has attracted considerable attention in 3D bioprinting areas because it has good biocompatibility, controllable biodegradability (depending on molecular weight and copolymer ratio), and has been approved by the US Food and Drug Administration (FDA) for human clinical usage [126,127].

The lack of suture resistance of 3D printed vascularized tissues and organs has always been an important problem hindering the development of organ manufacturing. If the anti-suturing ability is insufficient, the cell-laden natural hydrogel can hardly withstand any additional mechanical load. In 2010, Professor Wang’s research group produced a tubular three-layer PLGA-cell/fibrinogen-PLGA construct [137]. The inner and outer layers are made of supportive PLGA with different pores, which play an important role in long-term structural stability, preventing excessive expansion during mechanical stimulation. The middle layer is a fibrin-encapsulated cell hydrogel, which provides accommodation for cells to proliferate, migrate, and differentiate inside. The study found that the three-layer sandwich structure can withstand the maximum axial stress of 1100 kPa during the tubular contraction and extension stages, which is significantly higher than human blood pressure. With the enhancement of mechanical properties by PLGA, the ASCs in the intermediate fibrin hydrogel are induced to differentiate into smooth muscle cells and arranged regularly under growth factor induction and dynamic conditions. This strategy is expected to be widely used in the field of complex organ manufacturing.

As PLA, PLGA has been widely used in hard tissue and organ 3D prinitng due to its excellent mechanical properties. Researchers have prepared a series of double-layer PLGA-integrated scaffolds to achieve simultaneous repair of osteochondral tissue. In the experiment, two scaffolds with different apertures were made, and then they were glued together to form an integrated scaffold. By checking the condition of the porous scaffold implanted with bone marrow mesenchymal stem cells (BMSCs) for 24 weeks. It is proven that the double-layer PLGA porous scaffold can promote the repair of osteochondral defects and has application potential in osteochondral organ engineering [138]. 

### 4.3. PU

PU is a group of linear segment polymers, consisting of oligodiol (i.e., soft segment) and organic (i.e., hard segment) units connected through urethane (–NH– (C= O)–O–). PU can be biodegradable or non-biodegradable, and in vitro degradation studies have shown that PEG-based PUs are more degradable than PCL-based ones. In terms of mechanical properties, the tensile properties of PU are mainly controlled by soft segments. In another word, the difference in the structure of soft segments provides significant changes in the properties of PU. Studies have shown that the mechanical properties of PCL-based polymers are higher [128]. Acrylate groups can be used as ultraviolet (UV) curing sites incorporated into thermal PU for 3D printing of cells. These PUs show potentials for various biomedical applications including organ manufacturing [129].

**Figure 5:**
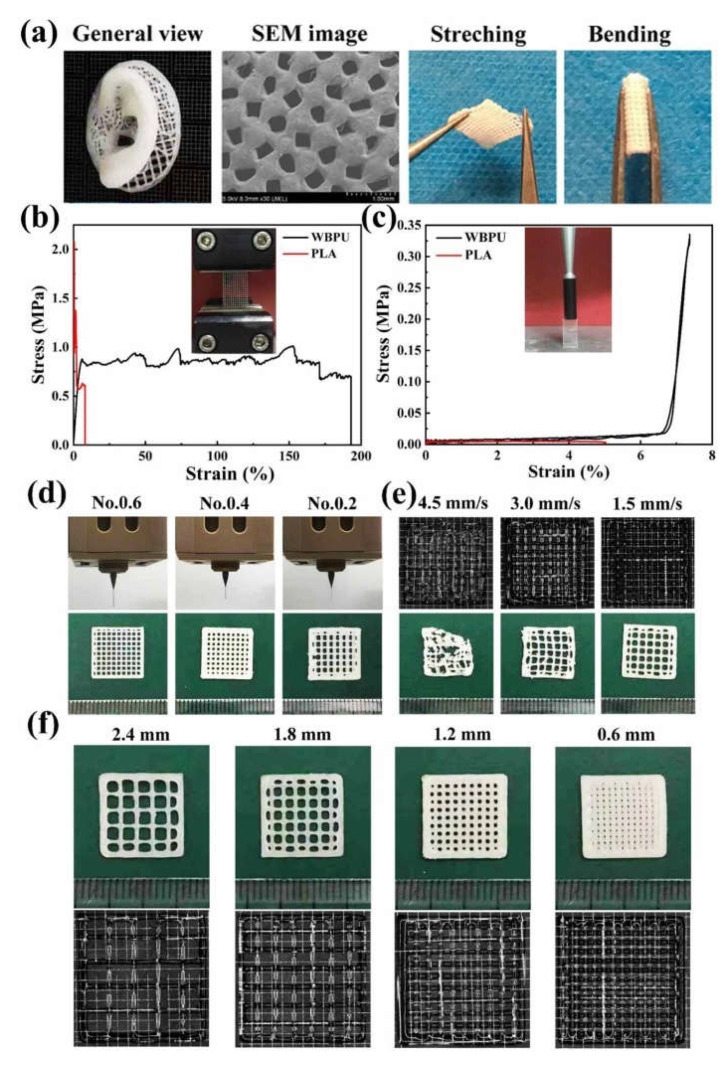
(**a**) A human ear model printed with WBPU; (**b**) Tensile stress–strain curves of the PLA and WBPU scaffolds; (**c**) Supporting force test results of the 3D printed PLA and WBPU scaffolds; (**d**) Using syringe needles of different models to print WBPU scaffolds; (**e**) 3D printing of micrographs of WBPU scaffolds at different extrusion speeds; (**f**) The micrographs of 3D printed WBPU scaffolds at different internal distance from strand [144].

Professor Wang of Tsinghua University developed a new biodegradable elastic PU, and carried out the 3D printing of various tissues and organs [93,139]. The biodegradable PU has been widely used in the fields of peripheral nerve repair conduits, rabbit vein recovery templates, and layered blood vessel/neural network overcoats [140,141]. In one of the experiments, Professor Wang’s research group used a dual-nozzle low-temperature deposition manufacturing (DLDM) system to prepare a double-layer polyurethane (PU)–collagen nerve repair conduits. By optimizing the process parameters and the polymer concentrations, an ideal nerve repair conduit was manufactured for peripheral nerve repair [142,143]. Feng et al. developed a biodegradable waterborne polyurethane (WBPU) modified by amino acid, which can be used by FDM 3D printing technology at a temperature of 50–70 °C. Through implantation experiments in mice, it is proven that the WBPU has good biocompatibility and degradability, and the degradation products have no cytotoxicity to host tissues (Figure 5) [144].

### 4.4. PCL

PCL, also known as polyε-caprolactone, formed by ring-opening polymerization of ε-caprolactone monomer catalyzed by a metal anion complex catalyst, is a biodegradable semi-crystalline polyester [130]. PCL is non-toxic, insoluble in water, and easily soluble in a variety of organic solvents. It has very good biocompatibility and biodegradability, which can be completely degraded in 6–12 months within a natural environment [131]. In addition, PCL also has temperature-dependent shape memory properties. Under heating conditions, it exhibits good viscoelasticity and rheology, and can be processed by FDM technology for four-dimensional (4D) printing.

Using extrusion-based 3D printing technologies PCL can be effectively printed into screw type stents with a controlled microstructure. The use of low-pressure nitrogen-based coatings can enhance cell adhesion and proliferation capacities without changing the mechanical properties of the stents [145]. In order to further enhance cell–cell signal transduction and cell–material interaction, Wang et al. used conductive biomaterials or mixed conductive fillers with non-conductive biomaterials for the manufacture of the electroactive PCL scaffolds [146]. Compared with traditional PCL and PCL/graphene scaffolds, the new scaffolds have improved in vitro biological performance because the nitrogen-doped graphene has higher conductivity and better surface hydrophilicity than the original graphene. Additionally, PCL lacks cell binding sites, the combination of PCL with natural polymers to create hybrid structures is a widely accepted strategy for PCL-based 3D bioprinting [147].

### 4.5. Pluronic Acid (or Poloxamer)

Pluronic acid (i.e. poloxamer) is a triblock copolymer composed of one hydrophobic poly (propylene oxide) (PPO) segment and two hydrophilic poly (ethylene oxide) (PEO) segments arranged in a PEO-PPO-PEO configuration; the general formula is: HO(CH4O)a(C3H6O)b(C2H4O)cH. The concentrated aqueous solution of poloxamer forms thermoreversible hydrogels. These hydrogels remain fluid at room temperature and become viscous once exposed to body temperature. The gel temperature is closely related to its concentration and structure (i.e., PPO/PEO ratio, PPO/PEO block length and, total polymer chain length) [132,133].

**Figure 6:**
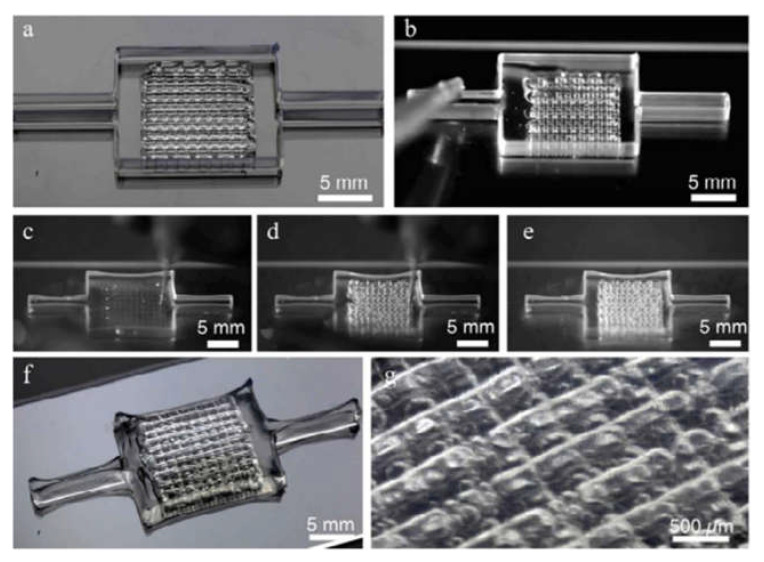
Highlight the image sequence used to make the printing, filling, and unorganized deinking process steps of the 3D vascular network embedded in the GelMa matrix: (**a**) Fugitive “ink” printed in the form of a 3D vascular network within silicone ink border; (**b**) Infilling of the 3D printed fugitive “ink” network with an acellular ECM of choice (GelMA shown); (**c**–**e**) Fugitive “ink” removal process at 4 °C; (**f**,**g**) Higher magnification images of the evacuated 3D vascular networks [149].

Pluronic acid shows good printability, but it has adverse effect on cell viability during long-term in vitro cultures, so it is often chemically modified with other polymers to improve the structural and mechanical properties [148,149]. Recently, researchers have combined pluronic acid with gelatin, which can take advantage of its excellent rheological behavior under shear stress and elasticity to create a biocompatible hydrogel for vascular channels. Müller et al. [150] reported a strategy for constructing nanostructured Pluronic hydrogels, which can significantly improve their biocompatibility. The mixed acrylate and Pluronic F127 can not only maintain the printability but also obtain a stable 3D structure through UV cross-linking. After 3D printing, the unreacted Pluronic was removed from the cross-linked network by elution. Methyl acrylate hyaluronic acid (HAMA) was also added to improve the mechanical properties of the 3D structures. There are some researchers chose 20% pluronic F127 aqueous solution to prepare biofilms [151]. After the neomycin-impregnated pluronic solution was mixed with polyvinyl alcohol (PVA) excipients (Povidon S630, PG) with appropriate concentration, the prepared neomycin-impregnated pluronic membrane inhibited the growth of bacteria on the agar plate through the sustained release of neomycin. When this pluronic membrane was implanted to the burn site of rabbits, it acts as a drug-releasing matrix with the potentials for local burntreatment (Figure 6).

## 5. Nanometer Material for Organ 3D Printing

In recent years, the application of two-dimensional nano materials (2DNM) in 3D printing has attracted extensive attention because of their special physical/chemical properties and excellent biocompatibilities. A series of nanomaterials, such as metal/metal oxide nanomaterials, are physically mixed or covalently bonded with polymer networks to produce speical biomedical devices, for the treatment of intractable diseases. 3D printing the multifunctional 2DNM “inks” to manufacture high-performance special organs is considered to be an emerging frontier in advanced material development and 3D bioprinting [152].

As a new type of crystalline and porous materials formed by metal nodes and polydentate ligands, metal organic frameworks (MOFs) have become a promising biomaterial in recent years because of their ultra-thin thickness, large surface area, and highly accessible active sites [153,154]. Nevertheless, most of the MOFs have poor water stability, which hinders their application in biomedical fields to a certain extent [155,156]. In this case, a large number of researchers have developed new “bioinks” with nano materials with high stability. For example, Ishiwata and his colleague used the cross-linking method to transform Zn based MOFs into polymer gel (PG) [157]. Colloidal particles or macromolecules in solution are connected with each other to form a spatial PG network. Additionally, the structural voids are filled with liquid. Tsotsalas et al. synthesized a copper free PG with excellent stability [158]. These gels can be used to adhere bacteria without releasing metal ions to ensure their applicability in biomedicine. Some metal/metal oxide containing ocomposite hydrogels exhibit not only enhanced mechanical properties, but also additional functions, such as electrical conductivity, magnetic properties, and/or antibacterial properties. The electrical activity of gold nanowire/alginate nanocomposite hydrogel makes it outstanding in special heart 3D printing with significantly increased electrical communication between adjacent cardiac myocytes, and stimulated synchronized contraction of cardiac myocytes through electrical stimulation [159]. It is predicted that this hydrogel will have great potential in cardiac tissue and organ manufacturing areas.

Many studies have shown that nano clay particles have important values in regenerative medicine, with respect to cell adhesion and diffusion, and controlled drug release [160]. Clay has been proved to be an excellent filler and can be designed as a new hydrogel for 3D bioprinting. The viscosity of polymer “bioinks” can be enhanced by nano clay increased, and nano clay can stabilize the polymer network [161,162]. Because of the reversible interaction between polymer and clay, the addition of synthetic clay improves the printability and shape fidelity of the alginate-based “bioinks”, changes the rheological properties of the gels, and makes the hydrogels more robust [163]. Furthermore, due to the low mechanical properties of natural polymers in 3D printing, “bioinks” based on nano-clay containing hydrogels have highlighted their advantages in 3D bioprinting areas, including increasable bioactivities, controllable mechanical prooperties, customizable degradation rates, and simple processing skills [164,165]. The PNAGA/nano clay hydrogel used for 3D bioprinting has achieved a good effect in repairing rat and rabbit bone defects [166].

Most inorganic ceramic nanoparticles contain some minerals naturally existing in the human body, which can provide biological functions to promote the growth of tissues in the body. Hydroxyapatite nanoparticles were incorporated into PEG hydrogel to form a tough elastic nanocomposite hydrogel, which supports osteoblast to adhere [167]. Compared with the PEG hydrogel alone, adding hydroxyapatite nanoparticles into PEG hydrogel can increase the toughness, breaking strength, and tensile modulus of the 3D printed structures. The further development of these elastic materials can promote the development of 3D printing in the manufacture of large tissues and organs.

## 6. Typical 3D Bioprinting Technologies for Bioartificial Organ Manufacturing

### 6.1. Heart 3D Bioprinting

Cardiovascular disease is one of the main causes of death in the world. Heart transplantation is the main method for many cardiovascular treatments at present. Recently, heart 3D printing technology has also made great progress and application.

3D bioprinting technology can be used to create heart valves. The commonly used printing materials for heart valves are biodegradable synthetic scaffolds and adult cells [168]. Laser sintering 3D printers are also used to make heart valve scaffolds, and human umbilical cord blood vessel cells can be planted on the scaffold [169].

3D bioprinting technology has been used to make myocardial chip platform. Some researchers use alginate, GelMA, and photoinitiator Irgacure 2959 as “bioinks” by encapsulating human endothelial cells, using coaxial extrusion printing to make a multilayer hydrogel microfiber scaffold, and then inoculating cardiomyocytes in the scaffold. Afterwards, the scaffold is further embedded in a specially designed microfluidic perfusion bioreactor to complete the endothelial myocardial chip platform for cardiovascular toxicity assessment, drug screening, and potential disease modeling [170].

3D bioprinting technology has been applied to create living heart tissues. Some researchers isolated the primary cardiomyocytes from the hearts of young rats and mixed them in the “bioinks” based on fibrin. They used the extrusion printing method to print the cell-laden hydrogels, sacrificed hydrogels, supportive PCL frames, and obtained cardiac tissues. The bioprinted heart tissues have spontaneous and synchronized contraction functions during in vitro cultures, and the progressive cardiac tissue development is confirmed by immunostaining of α-actinin and connexin. These results indicate that the bioprinted heart tissues are functional and can be applied in the fields of organ manufacturing and drug screening [171].

**Figure 7:**
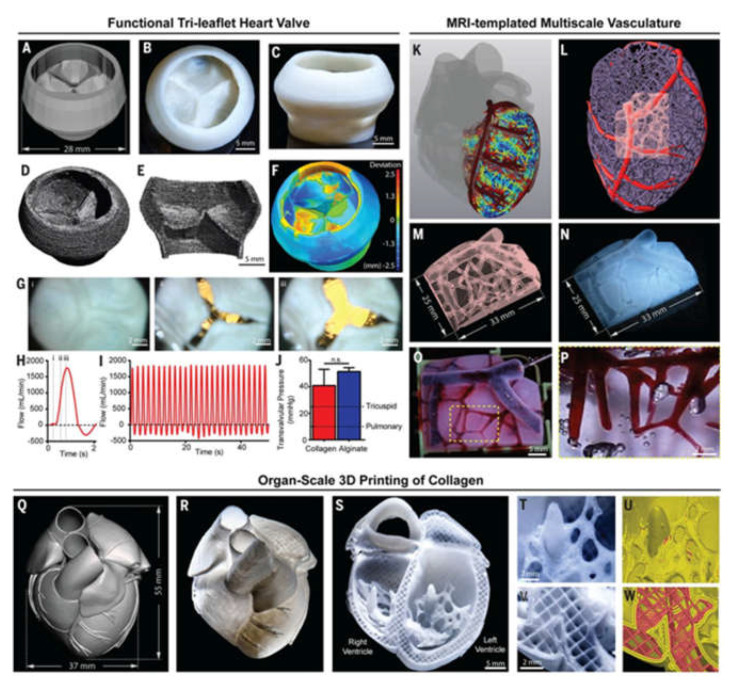
Organ-scale FRESH 3D bioprinting of tri-leaflet heart valve, multiscale vasculature, and neonatal-scale human heart: (**A**) Tri-leaflet heart valve 3D model at adult human scale; (**B**,**C**) Top and side views of FRESH-printed collagen heart valve; (**D**) Using μCT reconstruction showed the complete printed valve; (**E**) Lateral cross section of the wall and leaflets; (**F**) Compared with the 3D model, quantitative gauging of the μCT 3D surface shows average overprinting of +0.55 mm and underprinting of −0.80 mm; (**G**) Sequence of valve opening under pulsatile flow over 1 s; (H) Doppler flow velocimetry of a single cycle: (i) closed, (ii) half-open, (iii) open; (I) Repeat the cycle in (**H**); (**J**) Maximum transvalvular pressure of printed alginate and collagen valves compared to operating pressure for native valves [*N* = 3, data are means ± SD, n. s. indicates *p* > 0.05 (Student t test)]; (**K**) MRI-derived 3D human heart model (gray) with computationally derived multiscale vascular network shown for the left ventricle. The left anterior descending coronary artery (red) is the template to guide the formation of smaller-scale vessels, which decrease in diameter according to distance from the coronary artery (red to blue); (**L**) Left ventricle with the left anterior descending artery (red), computationally generated vasculature (purple), and subregion of interest (pink); (**M**) Transparent subregion showing 3D structure of the vascular network; (**N**) FRESH-bioprinted with collagen, showing reproduction of the vascular network; (**O**) The vascular network was perfused with red glycerin; (**P**) The collagen was optically removed and perfused with red glycerol to a diameter of 100-μm blood vessels; (**Q**) MRI-derived 3D human heart scaled to neonatal size; (**R**) FRESH-printed collagen heart; (**S**) A printed cross-sectional view of the collagen heart showing the left and right ventricles and the internal structure; (**T**,**U**) High-fidelity image of the trabeculae in the left ventricle (**T**) showing reproduction of the complex anatomical structure from the G-code (**U**); (**V**) A high-definition image of the septal wall between ventricles (**W**) Showing reproduction of the square-lattice infill [173].

3D bioprinting technology has also be used to create 3D heart models. Maiullari et al. combined human umbilical vein endothelial cells (HUVEC), induced pluripotent cell-derived cardiomyocytes, and encapsulated them in alginate and PEG–fibrinogen hydrogels. The “bioinks” are printed through a customized microfluidic printhead, and the 3D heart tissue consisting of iPSC derived CMs is obtained, which has defined geometric shapes generated by HUVEC. During the in vivo experiment, the host vascular system can penetrate and integrate into the bioprinting construct to provide rapid blood supply for the implant [172]. In addition, researchers have proposed a method of 3D bioprinting collagen using freeform reversible embedding of suspended hydrogels. By means of pH-driven gelation control, high resolution and porous microstructure can be achieved, and human heart components with various scales from capillaries to whole organs can be designed and printed (Figure 7) [173].

Nevertheless, the structure of the natural heart is extremely complex, and the complicated blood vessels, neural networks, and muscle tissues in the heart are unusually difficult to construct at the same time under the existing 3D bioprinting technologies [174]; whether the 3D bioprinted constructs can realize the function of the heart biophysically and biochemically remains to be further explored [175].

### 6.2. Liver 3D Bioprinting

The liver is composed of highly specialized tissues that play an important role in metabolism and have multiple functions, including regulation of glycogen storage, red blood cell breakdown, plasma protein synthesis, hormone production, and detoxification. Anatomically, the liver is divided into four lobes, and each lobe is composed of numerous liver lobules under the microscope. The lobules are roughly hexagonal in shape and consist of hepatocyte plates radiating from the central vein. There are two main types of liver cells: parenchymal cells and non-parenchymal cells. Parenchymal cells are hepatocytes, accounting for 70–85% of the liver volume. Non-parenchymal cells include sinusoidal endothelial cells (SEC), phagocytic Kupffer cells (KC), and hepatic stellate cells (HSC) [176,177,178]. These non-parenchymal cells play special roles in certain physiological functions. For example, HSC is heavily involved in the synthesis of growth factors and the regeneration of ECM proteins, both of which play a key role in hemostasis and cell signaling [179].

Since hepatocytes have high proliferation capacity, the liver has extensive regeneration capacity. However, due to the limited availability of adult hepatocytes, they are difficult to isolate, and possess poor reproductive ability and rapid degradation of in vitro functions [180]. Various techniques have been developed to produce bionic liver tissues. However, traditional methods are unable to break through the existing bottleneck in producing volumetric liver tissue with high intercellular adhesion.

At the same time, due to its high fidelity and the ability to quickly and accurately manufacture relatively complex 3D structures, 3D bioprinting can manufacture more complex tissues and organs. Therefore, in recent years, 3D bioprinting technology has been increasingly used to manufacture complex liver structures with higher cell density [181,182].

**Figure 8:**
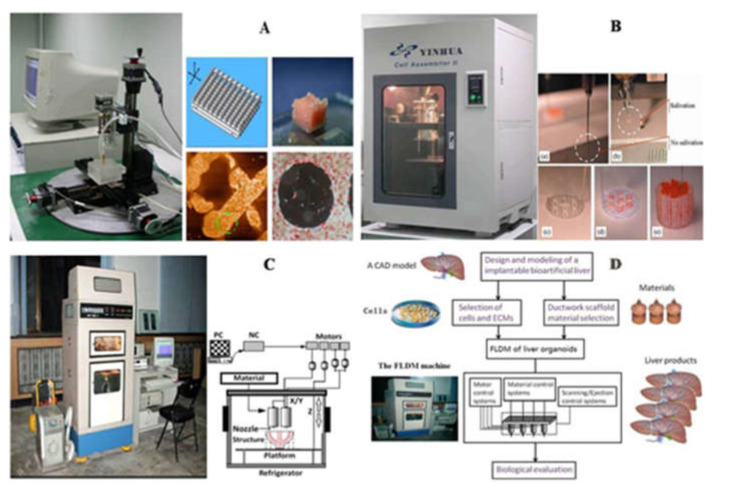
Schematic diagrams of Tsinghua University, Professor Wang, laboratory produced several 3D bioprinters: (**A**) in 2004, gelatin hydrogel was printed to large living tissue through single nozzle 3D biological printer; (**B**) in 2007, two types of cells in gelatin-based hydrogels were printed into large organs; (**C**) was used to print cell-laden gelatin-based hydrogels and synthetic polymers with branched vascular networks; (**D**) in 2010, liver modeling and manufacturing was carried using a four nozzle low-temperature 3D printer [12].

Using RP technology, the research group of Professor Wang of Tsinghua University has successfully manufactured various hepatic tissues and organs using hepatocytes and gelatin-based hydrogels. Professor Wang has conducted a series of groundbreakings using a series of self-made 3D printers (Figure 8). In one study, a highly accurate 3D micro-positioning system with a pressure-controlled injector was created to deposit cells/hydrogels with a lateral resolution of 10 microns, creating various 3D patterns with different channel arrays (or go-through holes). More than 30 layers of hepatocytes/hydrogels were printed into a high-space constructs. Hepatocytes remained viable in the constructs and perform biological functions for more than 2 months [71]. In another study, a double-nozzle bioprinting technology was used to create an anatomical liver structure with a blood vessel-like networks. Adipose-derived stem cells (ASCs) wrapped in a gelatin/alginate/fibrinogen hydrogel were printed to form the blood vessel-like networks, hepatocytes were loaded with gelatin/alginate/chitosan hydrogel around it, and endothelial growth factor was used to induce the ASCs to differentiate into endothelial-like cells. The results show that the albumin secretion level of the embedded hepatocytes is cultured for 2 weeks, but the levels of urea and alanine aminotransferase decreased after the increase. These results indicate that this dual-nozzle 3D printing technology can be a powerful tool for manufacturing complex liver structures with special internal/external structures. In order to better realize the differentiation of stem cells and the assembly of different cell types, Professor Wang has pioneered a combined four nozzle 3D printer to quickly produce bioartificial livers. In other research groups, researchers demonstrated the application of 3D digital bioprinting technology to create a 3D hydrogel-based ternary culture model that uses gelatin methacrylate and glycidyl methacrylate–hyaluronic acid to derive iPSC hepatic progenitor cells, human umbilical vein endothelial cells and ASCs are embedded in a miniature hexagonal structure. The results showed that the morphology of the model was improved, the expression level of liver-specific genes was higher, the secretion of metabolites increased, and the induction of cytochrome P450 was enhanced [183].

### 6.3. Neural 3D Bioprinting

Currently, more than 50 million people worldwide suffer from neurodegenerative diseases, and most of the existing treatment methods for neurodegenerative diseases, as well as acute traumatic injury are exceedingly restricted due to the adult neurons are hard to divide and proliferate. In order to repair the neural tissues, various 3D bioprinting technologies have been employed to make nerve tissues with physiological functions [184].

Some researchers embedded neural stem cells (NSCs) in thermally responsive biodegradable PU “bioinks”. The thermally responsive and biodegradable PU “bioinks” were suitable for 3D printing at around 37 °C without any cross-linking [185]. The NSC embedded in the water-based PU hydrogel with appropriate stiffness showed considerable vigor and differentiation capability after 3D printing. When the 3D printed construct was implanted, the function of adult zebrafish suffering from traumatic brain injury was restored. It is an effective method for future nerve tissue restoration. Studies have shown that it is possible to construct neural tissues by printing human NSCs that support the production of functional neurons and glial cells in-situ [186]. The “bioinks” used in the study include stem cell-containing alginate, carboxymethyl chitosan, and agarose. The printed “bioinks” quickly gel through stable cross-linking to form a 3D object, which encapsulates the stem cells for in-situ expansion and differentiation. The results of experiment indicate that the differentiated neurons formed synaptic contacts, established a network with increased calcium response induced by bicuculline. Wang et al. have 3D printed a poly(3,4-ethylenedioxythiophene)–chitosan–gelatin (PEDOT–Cs–Gel) scaffold through in-situ interfacial polymerization. They assembled a PEDOT nanostructure layer on the channel surface of the porous Cs–Gel scaffold to make a conductive PEDOT–Cs–Gel scaffold. The scaffold was used for 3D culture of NSCs in vitro, and it was found that the PEDOT layer on the surface of the Cs–Gel scaffold channel can greatly promote the adhesion and proliferation of NSCs. In addition, this scaffold can significantly enhance the differentiation of NSCs into neurons and astrocytes by up-regulating the expression of β tubulin-III and GFAP [187]. 

Since the differentiation of NSCs can be oriented using biologically active macromolecules and transcription factors, in order to make cells differentiate accurately, it is necessary to create 3D cell survival environments with precise spatial patterns. Some researchers have directly patterned nerve cells in a 3D multilayer collagen gel [188]. They print a layer of collagen precursor to provide a scaffold for the cells, then print the rat embryonic neurons and astrocytes on this layer, and finally apply the sodium bicarbonate solution as aerosolized aerosol to the cells containing the collagen layer. This makes the collagen gel, and finally builds a 3D cell–hydrogel composite [189].

Additionally, extrusion-based 3D bioprinting models have greater advantages and potentials in neural drug screening and brain cancer treatments compared to traditional 2D cell cultures. Various neural growth factors (GFs) can be added to the 3D printing hydrogels directly and released over time. The 3D printing technology is widely considered to be more helpful to promote the embedded cells/GFs/drugs to migrate and communicate [190]. A 3D bioprinted glioma stem cell model has been established using a modified gelatin/alginate/fibrinogen hydrogel that mimics the ECM. Glioma stem cells show high activity with increased proliferation rate, glial fibrillary acidic protein and β-tubulin III expression [100].

### 6.4. Skin 3D Bioprinting

The skin is the largest organ of the human body [191], and it is a complex multi-layered structure composed of multiple cellulars, ECM fibers, small veins, capillaries, nerves, and hair follicles. The skin of humans is composed of three layers: epidermis, dermis, and hypodermis [192].

At present, there are two main strategies for skin regeneration. One is to inoculate cells on a degradable scaffold, and then induce a mature 3D tissue structure; the other is to encapsulate the cells in “bioinks” through a layer-by-layer print to obtain complex 3D skin tissues [3].

**Figure 9:**
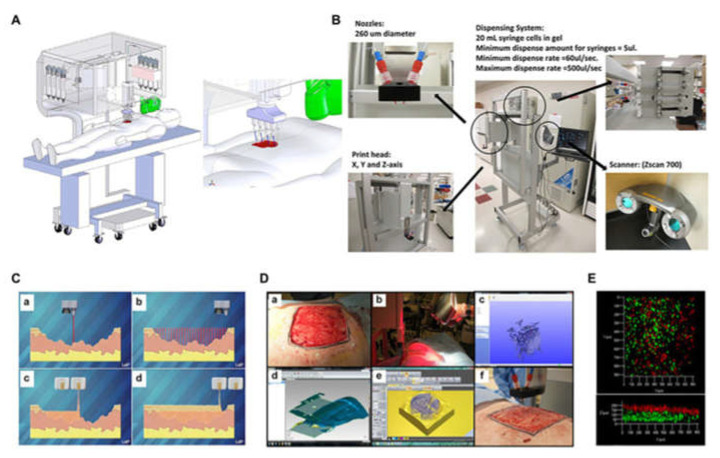
Skin bioprinter prototype and in situ bioprinting concept: (**A**) Design and component diagram of skin bioprinter; (**B**) The main components of the system include a nozzle with a diameter of 260 µm, eight independent distribution systems to drive the print head connected to the XYZ mobile system, and a 3D wound scanner; (**C**) Skin bioprinting. The specific information of the defect wound is obtained by scanning, and then the specified materials and cells are deposited in the appropriate position through the print nozzle; (**D**) Example of skin bioprinting process (**a**) markers placed around the wound are used as reference points; (**b**) Before scanning with the handheld Z700 scanner; (**c**) Then, the geometric information obtained by scanning is input in the form of STL file to orient the scanned image to the standard coordinate system; (**d**) the scanning data and its coordinate system are used to generate the filling position and nozzle head moving path to fill the defect model; (**e**,**f**) the output code is then provided to the custom bioprinter control interface to generate the nozzle path; (**E**) required to print the fill volume. The system can control the deposition of multiple cell types. Shows stratification of fibroblasts (green) and keratinocytes (red) [196].

Some researchers use keratinocytes and fibroblasts as the constituent cells representing epidermis and dermis, and collagen is used as the dermal matrix representing skin. By inkjet printing on demand, collagen layer and single cell layer are printed separately to construct a 3D skin tissue. Histological and immunofluorescence experiments show that 3D printed skin tissue is similar to human skin in morphology and biology, and can be used as a model for studying pathophysiology of skin diseases [193]. Laser-assisted bioprinting technology is also used to create a fully cellular skin substitute, by placing fibroblasts and keratinocytes in a stable matrix, and implanting them in mice. The resulting skin structure can be completely connected to surrounding tissues, and some blood vessels can grow from the edge of the wound to the printed cells [194]. Some researchers also used extrusion bioprinters to first print “bioinks” containing human foreskin dermal fibroblasts (hFB), endothelial cells (hECs), and placental pericytes (hPC) suspended in mouse tail type I collagen to form the dermis and then print “bioinks” containing human foreskin keratinocytes (hKC) to form the epidermis. When the bioartificial skin tissues are implanted into the back of immunodeficient mice, the hEC lining structure gradually merged with the mouse capillaries from the wound bed [195]. In recent years, some researchers have proposed a novel design of mobile skin bioprinting system. The system integrated imaging technology with 3D inkjet bioprinting technology, wound data, and transported different types of cells to specific locations in-situ to achieve rapid repair of large-area wounds (Figure 9) [196].

Bioprinting technology is of special significance for skin organ construction. Ulcers or burns cause extensive skin defects. Autologous skin transplantation is limited, and a large amount of autologous skin can be obtained through bioprinting technology. However, at present, there is still some distance for the constructed skin to be used in clinic, partly due to the complexity of the multi-layer skin tissues. It is expected that with the rapid development of 3D printing technologies, large scale-up skin manufacturing with improved biological functions will come true in the near future [197].

## 7. Challenges

As discussed in this review, 3D bioprinting technology has made significant research progress in complex organ manufacturing using various polymeric “bioinks”. However, there are still many challenges in the manufacture of clinical useful large bioartificial organs. As organ manufacturing is an interdisciplinary subject and the result of the integration of biology, chemistry, physics, informatics, computer, and medicine, it is necessary to integrate talents in a wide range of fields such as biology, materials, chemistry, physics, machinery, medicine, and clinic to solve these challenges [198,199,200].

With the increase in the structure and size of the 3D printed organs, the transportation of nutrients is becoming more and more evident. In endogenous organs, nutrients/oxygens are transported through the vascular networks. The organs produced by 3D printing need to anatomically imitate the vascular networks in natural organs; provide water, gas, and nutrients for cells; and discharge metabolites from cells, which is the key to prevent organ necrosis [201,202,203,204,205]. In order to form highly simulated organs on a macro scale, a large amount of cells is needed before 3D printing. It is preferred to use stem cells with the capability to proliferate and differentiate into the target cell types as the cell sources. Unremitting efforts of biological experts are needed to solve the pertinent problems in this field.

The mainstream of the future development of 3D bioprinting is the use of multi-nozzle 3D printers for organ manufacturing. Only by using multi-nozzle printers can we assemble as many homogeneous and heterogeneous cells and useful polymers as possible. Therefore, mechanical engineers must innovate or update the 3D organ printers and solve the engineering related problems, such as the insufficient printing resolution, the slow printing speed, the instability of “bioinks”, and the standardability of nozzles [206,207]. Only by solving these problems in advance can we create more complex organ substitutes that can meet all the clinical requirements.

The organ made by bioprinting is a bionic structure, so the compatibility between the cell-laden polymers and cells must be considered at first. It has certified that any single polymer and cell type cannot have all the characteristics required for a bioartificial organ manufacture. Therefore, it is a compelling obligation for chemists and/or material scientists to develop more appropriate “bioinks” for different organ manufacturing [15,201].

Finally, in the process of organ implantation, doctors need to ensure the structural integrity and mechanical property (including stiffness) of the bioartificial organs, realize the physiological functions of the target organs, and ensure no syndromes after operation [28].

## 8. Conclusions

At present, it is possible to manufacture bioartificial organs including layered blood vessels and neural networks. Although the internal vascular networks in printed organs and the in-situ controlled differentiation of stem cells to more than three types of cells are still the main obstacles for 3D bioprinting large scale-up complex organs, we can still foresee that 3D bioprinting technology has broad clinical potentials in organ manufacturing both for bulk production and personalized treatment, and the production of homogeneous allogeneic and autologous organs will inevitably move from the field of science fiction to reality, and the 3D printing based organ manufacturing will beyond all doubt push the precision medicine to a new level.

## Figures and Tables

**Table 1:** Comparation of 3D bioprinting technologies for organ manufacturing.

Printing Technology	Principle	Material	Advantages	Defects	Ref.
Inkjet 3D printing	Unsing acoustic, thermal or piezoelectric nozzle to jet biomaterials in drops	Polymer solutions or cell suspensions	Efficient control of “bioinks”, low cost and high throughput	Biomaterials need to be in a liquid state, and the viscosity needs to be precisely controlled	[17,18,19]
Fused deposition modeling	Thermoplastic material melted through one or more heated extrusion heads with small holes in a specific laying method	Thermoplastic polymers with a certain viscosity after heating, such as PCL, PLA, ABS, etc.	Low cost, a wide range of nonbiodegradable synthetic polymers with excellent mechanical properties can be printed	High printing temperature in which cells, growth factors and other bioactive agents cannot be incorporated	[20,21,22,23,24,25,26]
Extrusion based 3D printing	Polymeric solutions or hydrogels are drawn, extruded and deposited to form solid structures	A variety of natural polymers, such as alginate, collagen, and chitosan can be selected	High accuracy and speed, cells and other bioactive agents can be incorporated	Some of the extrusion setups may cause damage to cells	[27,28,29,30,31,32,33,34,35]
Stereolithography	Laser or projected light converts the liquid photosensitive material into a solid platform	Only photosensitive polymers can be used	High accuray	Most of the photosensitive resins are toxic to cells and the light in the printing process affect the survival rate of the cells	[36,37,38,39,40,41]
Aerosol jet printing	Ultrasonic or pneumatic atomization is formed by squeezing the “bioinks” around the airflow	Any substance that can be suspended in an aerosol	High resolution and flexibility, it can be printed on various substrates such as metals, semiconductors, and polymers	Denature DNA	[42,43,44,45,46]

**Table 2:** Commonly used natural polymers for 3D bioprinting.

Polymer	Chemistry	Characteristic	Disadvantage	Ref.
Gelatin	Partial degraded product of collagen	Excellent biocompatibility	Unstable solution at room temperature, fast degradation rate, and poor mechanical property	[51,52]
Alginate	A linear anionic polysaccharide copolymer	Rich source, low price, good hydrophilic property, easy to form penetrating networks	Few cells attachment sites and fast degradation rate	[53,54]
Fibrin	Polymerization product of fibrinogen	Excellent biocompatibility and biodegradability	Poor long-term stability and mechanical strength	[55,56,57]
Hyaluronic acid	A linear high molar mass natural polysaccharide	Non-allergic and non-inflammatory	Fast degradation rate and poor mechanical property	[58,59]
Collagen	A kind of protein composed of three intertwined polypeptide chains, which are connected to each other by hydrogen bonds and covalent bonds	In vivo immunogenicity, good cell compatibility	Poor mechanical properties	[60,61]
Silk fibroin	A natural protein from insects	Good biocompatibility and mechanical properties	Slow degradation rate	[62]
Chitosan	Obtained through deacetylation of chitin	Good biocompatibility, biodegradability, cell adhesion capability, low cost	Poor mechanical strength, unstable gel state	[63,64]
Agarose	Linear polysaccharide	Slow degradation rate, low cost, good mechanical properties after gelling	Poor cell compatibility	[65]

**Table 3:** Gelatin-based polymers for organ 3D printing.

3D Bioprinting Technique	“Bioink” Formulation	Crosslinking Method	Application	Ref.
One nozzle extrusion-based 3D bioprinting	Hepatocytes in gelatin/chitosan hydrogel	3% sodium tripolyphosphate (TPP)	Hepatic tissue manufacturing	[70]
	Hepatocytes in gelatin hydrogel	2.5% glutaraldehyde	Hepatic tissue manufacturing	[71]
	Hepatocytes in gelatin/fibrinogen hydrogel	Thrombin induced polymerization	Hepatic tissue manufacturing	[72]
	Gelatin/hyluronan	2% glutaraldehyde	Brain tissue repair	[73]
Two-nozzle low-temperature extrusion-based 3D printing	Polyurethane (PU)-gelatin/5% or 10% lysine hydrogel	0.25% glutaraldehyde	Liver manufacturing	[74]
	PU-adipose-derived stem cell (ADSC)/gelatin/alginate/fibrinogen/glycerol or dimethyl sulfoxide (DMSO) hydrogel	Double crosslinking with CaCl_2_ and thrombin solutions	Bioartificial liver manufacturing	[75]
One-syringe extrusion-based 3D printing	Nanosilicate/GelMA	UV light (320–500 nm) for 60 s at an intensity of 6.9 mW/cm^2^	Electrical conductive agent for bone tissue engineering	[76]
EnvisionTEC 3D-Bioplotter^®^	Polyethylene glycol (PEG)/gelatin-PEG/fibrinogen	Gelatin scaffolds were cross-linked with 15 mM EDC and 6 mM NHS, fibrinogen-containing samples were treated post-printing with 10 U/mL thrombin in 40 mM CaCl_2_ for ~30 min	Grid structures for cell seeding	[77]
Dual-syringe Fab@Home printing device	Gelatin ethanolamide methacrylate (GE-MA)-methacrylated hyaluronic acid (HA-MA) (GE-MA-HA-MA)/HepG2 C3A, NIH 3T3, or Int-407 cell	Ultraviolet (UV) light (365 nm, 180 mW/cm^2^) photocrosslinking	Tubular hydrogel structures for cell attachment	[78]
EHD inkjet printing system	GelMA solution	Illuminating with a UV light source	Microvascular constructs	[79]

**Table 4:** Alginate-based polymers for organ 3D printing.

3D Bioprinting Technique	“Bioink” Formulation	Crosslinking Method	Bioprinter	Ref.
One/two nozzle extrusion-based 3D bioprinting	GelMA/alginate/PEGTA	Photo-crosslinking/ CaCl_2_ solution	Novogen MMX Bioprinter™	[87]
	Alginate/chitosan hydrogel	CaCl_2_ solution	EFD^®^ Nordson printer	[88]
	Nanocellulose-alginate	CaCl_2_ solution	3D discovery printer	[89]
	Zinc oxide (ZnO) nanoparticles (NPs)/alginate	CaCl_2_ solution	BioBot 1	[90]
	Propolis/sodium alginate	CaCl_2_ solution	Ultimaker^2+^	[91]
	Sodium alginate/keratin	CaCl_2_ solution	Ultimus V	[92]
Inkjet-based 3D bioprinting	Collagen/sodium alginate	CaCl_2_ solution	HP DeskJet 550C	[93]
	Alginate solution	CaCl_2_ solution as substrate	SEA-Jet™	[94]
	Sodium alginate solution	CaCl_2_ solution	A platform-assisted 3D inkjet bioprinting system	[95]
	Alginate solution	CaCl_2_ solution after printing	MicroFab MJ-ABL piezoelectric inkjet printhead printer	[96]
One/two-syringe extrusion-based 3D printing	Gelatin/glucose-alginate hydrogel	CaCl_2_ solution after printing	Fab^@^Home Model1-3	[97]
Laser-based bioprinting	Sodium alginate/ Nano-HA	Laser	BioLP workstation	[98]
	Sodium alginate solution	CaCl_2_ solution	Matrix-assisted pulsed-laser evaporation direct-write	[99]

**Table 5:** Commonly used synthetic polymers for organ 3D printing.

Polymer	Chemistry	Characteristic	Ref.
PLA	A linear thermoplastic aliphatic polyester, mainly produced from starch raw materials through saccharification, fermentation and other chemical reactions	Good biocompatibility and biodegradability, can be completely degraded under certain conditions	[122,123,124,125]
PLGA	A synthetic copolymer of lactic acid (LA) and glycolic acid (GA), synthesized by the ring-opening copolymerization of cyclic dimer (1,4-dioxane-2,5-dione), glycolic acid and lactic acid	Good biocompatibility and controllable biodegradation rate	[126,127]
PU	A set of linear units (–NH–(C=O)–O–) connected by oligodiol (i.e., soft segment) and organic (i.e., hard segment) units through carbamate (i.e., carbamate)	Controllable degradation rate and mechanical properties, can be modified to have heat-sensitive properties	[128,129]
PCL	Catalysis with metal anion complex catalyst ε- Formation of caprolactone monomer by ring opening polymerization	Good biocompatibility and biodegradability	[130,131]
Pluronic Acid	Compound with a basic structure of poly (ethylene oxide) (PEO)-poly (propylene oxide) (PPO)-PEO	Easy to prepare, good cell affinity, and heat-sensitive	[132,133]

## Data Availability

Not applicable.

## Abbreviations

**Abbreviations**	**Full name**
3D	Three-dimensional
RP	rapid prototyping
AM	additive manufacturing
SFM	solid free-form surface manufacturing
CAD	computer-aided design
FDM	fused deposition modeling
PEGDA	PEG-diacrylate
GAG	glycosaminoglycans
PMC	polymer matrix composites
PCC	polymer ceramic composites
FRC	fiber reinforced composites
ABS	acrylonitrile butadiene styrene
PLA	polylactic acid
ASA	acrylonitrile styrene acrylate
PC	polycarbonate
PEEK	polyether ether ketone
ULTEM	polyether imide
HA	hyaluronic acid
AHA	aldehyde hyaluronic acid
CMC	N-carboxymethyl chitosan
GEL	gelatin
ALG	alginate
SLA	Stereolithography
GelMA	gelatin-methacryloyl
HAMA	methacrylated hyaluronic acid
SMC	smooth muscle cells
VIC	valve interstitial cells
TPP	tripolyphosphate
ZnO	zinc oxide
NPs	nanoparticles
EDC	1-Ethyl-3-(3-dimethylaminopropyl) carbodiimide
PEG	Polyethylene glycol
NHS	N-hydroxysuccinimide
PBS	phosphate buffered saline
ACGO	agarose/chitosan/graphene oxide
PGA	glycolic acid
PLGA	polylactic-co-glycolic acid
PU	polyurethane
LA	lactic acid
GA	glycolic acid
FDA	Food and Drug Administration
BMSCs	bone marrow mesenchymal stem cells
UV	ultraviolet
DLDM	dual-nozzle low-temperature deposition manufacturing
WBPU	waterborne polyurethane
PPO	propylene oxide
2DNM	two-dimensional nano materials
MOF	Metal Organic Frameworks
PG	polymer gel
iPSC	induced pluripotent cell
CMs	cardiomyocytes
HUVEC	Human Umbilical Vein Endothelial Cells
SEC	sinusoidal endothelial cells
KC	Kupffer cells
HSC	hepatic stellate cells
ASCs	adipose-derived stem cells
NSC	neural stem cells
PEDOT	poly(3,4-ethylenedioxythiophene)
Cs	chitosan
GF	growth factor
ECM	extracellular matrix
FB	fibroblasts
EC	endothelial cells
